# The role of the cerebellum in adaptation: ALE meta‐analyses on sensory feedback error

**DOI:** 10.1002/hbm.24681

**Published:** 2019-06-02

**Authors:** Joseph F. Johnson, Michel Belyk, Michael Schwartze, Ana P. Pinheiro, Sonja A. Kotz

**Affiliations:** ^1^ Maastricht University Maastricht the Netherlands; ^2^ Bloorview Research Institute Holland Bloorview Kids Rehabilitation Hospital Toronto Canada; ^3^ Faculdade de Psicologia ‐ Universidade de Lisboa Lisboa Portugal; ^4^ Max Planck Institute for Human Cognitive and Brain Sciences Leipzig Germany

**Keywords:** cerebellum, fMRI, forward model, meta‐analysis, prediction, sensory feedback

## Abstract

It is widely accepted that unexpected sensory consequences of self‐action engage the cerebellum. However, we currently lack consensus on where in the cerebellum, we find fine‐grained differentiation to unexpected sensory feedback. This may result from methodological diversity in task‐based human neuroimaging studies that experimentally alter the quality of self‐generated sensory feedback. We gathered existing studies that manipulated sensory feedback using a variety of methodological approaches and performed activation likelihood estimation (ALE) meta‐analyses. Only half of these studies reported cerebellar activation with considerable variation in spatial location. Consequently, ALE analyses did not reveal significantly increased likelihood of activation in the cerebellum despite the broad scientific consensus of the cerebellum's involvement. In light of the high degree of methodological variability in published studies, we tested for statistical dependence between methodological factors that varied across the published studies. Experiments that elicited an adaptive response to continuously altered sensory feedback more frequently reported activation in the cerebellum than those experiments that did not induce adaptation. These findings may explain the surprisingly low rate of significant cerebellar activation across brain imaging studies investigating unexpected sensory feedback. Furthermore, limitations of functional magnetic resonance imaging to probe the cerebellum could play a role as climbing fiber activity associated with feedback error processing may not be captured by it. We provide methodological recommendations that may guide future studies.

## 1 INTRODUCTION

**Figure 1:**
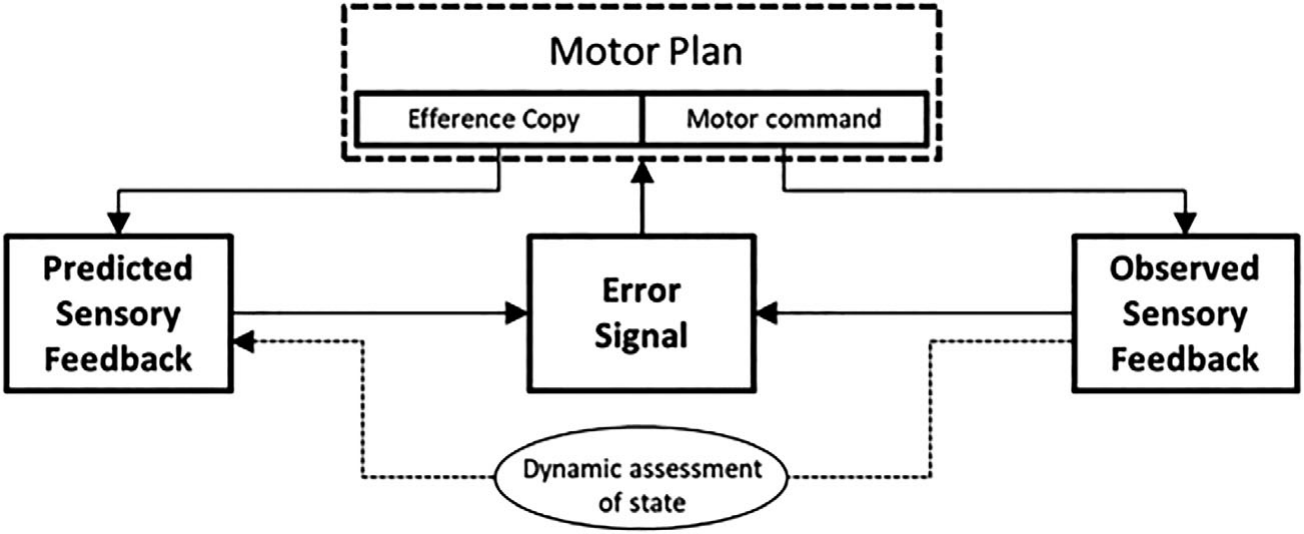
Components of a forward model. A diagram which outlines five major components of a Forward Model. The “Motor Plan” incorporates the components (1) “Efference Copy” and (2) “Motor Command.” The implantation of the Motor Command leads to (3) “Observed Sensory Feedback.” The efference copy is an expectation of sensory consequences of the enactment of the motor plan, providing (4) “Predicted Sensory Feedback.” The Observed Sensory Feedback and the Predicted Sensory Feedback are compared. If they do not match, an (5) “Error Signal” indicating violation of the expected consequences is returned for updating of the motor plan. The “Observed Sensory Feedback” can also notify the “Predicted Sensory Feedback” with contextual information of body or environment in order to make temporary changes to the prediction rather than updating the motor plan. This is denoted as “Dynamic assessment of state”

To successfully act within a dynamic environment, we continuously monitor sensory feedback associated with our own movements to ensure our actions have the desired outcomes. Even the simplest movements require complex coordination between multiple effectors. The continuous monitoring of sensory feedback helps to refine motor plans and adjust them to contextual and environmental changes. The forward model is a computational process that compares expected to actual sensory consequences of an action (see Figure 1; Jordan & Rumelhart, 1992; Miall & Wolpert, 1996; Wolpert, 1997). This comparison is essential to motor control and relies partly on the cerebellum (Blakemore, Frith, & Wolpert, 2001; Ishikawa, Tomatsu, Izawa, & Kakei, 2016; Ito, 1984a; Kawato, Furukawa, & Suzuki, 1987; Miall, Weir, Wolpert, & Stein, 1993; Wolpert, Miall, & Kawato, 1998). However, there is still no strong consensus on where in the cerebellum unexpected sensory feedback is processed. To this end, the current meta‐analysis systematically explores patterns of cerebellar activation in neuroimaging studies of sensory feedback manipulations. Such manipulations create an artificial mismatch between intended and perceived sensory consequences of an action, constituting methods commonly used to probe forward models in brain imaging studies.

A fundamental component of the forward model is the *efference copy*: when the motor cortex sends a command to the peripheral nervous system for the execution of motor behavior, a copy of the command is fed forward to provide an estimate of the sensorimotor feedback predicted from the movement (von Holst & Mittelstaedt, 1950). The predicted feedback is compared to the actual feedback from the proprioceptive (Miall & Wolpert, 1996; Wolpert et al., 1998), visual (Leube et al., 2003), auditory (Hashimoto & Sakai, 2003), and tactile (Blakemore, Wolpert, & Frith, 1998) sensory periphery. Any discrepancy between predicted and actual feedback constitutes an output known as a corollary discharge (Feinberg, 1978). The resultant error signal is processed by the cerebellum, ultimately resulting in an output to cortical areas to adaptively fine‐tune behavior. As both the state of the organism and the environment are dynamic, forward models are employed continuously to reduce any discrepancy between predicted and actual feedback through constant monitoring and adjustment of behavior (Desmurget & Grafton, 2000; Jordan & Rumelhart, 1992).

The cerebellar cortex comprises overlapping functional zones that process input from specific sensory modalities (Witter & De Zeeuw, 2015). Specific areas respond to auditory or visual stimuli (O'Reilly, Beckmann, Tomassini, Ramnani, & Johansen‐Berg, 2009; Petacchi, Laird, Fox, & Bower, 2005; Sang et al., 2012). Likewise, portions of the cerebellum segment into somatomotor topographies associated with the control of different body parts (Buckner, Krienen, Castellanos, Diaz, & Yeo, 2011; Mottolese et al., 2012). These divisions are coupled to associated subdivisions of motor cortex responsible for controlling the same body parts. The motor areas contribute cortical input to communication loops between the cortex and cerebellum employed in ongoing motor control. Therefore, the cerebellar cortex receives two principle types of afferents: mossy fibers primarily via the pons which relay information such as the efference copy from corresponding cortical regions (Raymond, Lisberger, & Mauk, 1996), while medullary nuclei relay bottom‐up sensory feedback‐related signals and induce changes in the influence of cortical top‐down signals to the cerebellar cortex (Ito, Sakurai, & Tongroach, 1982). The inferior olive monitors the discrepancy between predicted and actual sensory input and relays an error signal to the cerebellum via climbing fibers (Ito, 1984b; Kawato & Gomi, 1992). The cerebellum also receives signals of unpredicted auditory feedback from the dorsal cochlear nuclei of the medulla (Schwartze & Kotz, 2013). In turn, the cerebellum continuously signals the discrepancy back to the cerebral cortex to induce adaptation of motor behavior until the expected feedback matches the actual sensory feedback.

In order to guide the complex task of adapting behavior to resolve discrepancy between expected and actual sensory feedback, the cerebellum must communicate and coordinate with multiple areas of the cerebral cortex. All output from the cerebellum, such as from the processing of the corollary discharge, are issued via deep cerebellar nuclei (Middleton & Strick, 1997), connecting to regions such as the prefrontal cortex (Allen et al., 2005; Balsters et al., 2010; Kelly & Strick, 2003; Middleton & Strick, 2001; Ramnani, 2012; Watson, Becker, Apps, & Jones, 2014), motor areas (Akkal, Dum, & Strick, 2007; Balsters et al., 2010; Dum & Strick, 2003; Kelly & Strick, 2003; Lu, Miyachi, & Takada, 2012; Wise & Strick, 1984), and parietal area (Allen et al., 2005; Clower, West, Lynch, & Strick, 2001; Prevosto, Graf, & Ugolini, 2009; Ramnani, 2012). The cerebellum and these frontal and parietal areas also create loops where reciprocal exchange of information can be continuously fed through a cortico‐cerebellar system to allow for continuous adaptation of motor activity (Watson et al., 2014).

A common experimental approach to study neural substrates that implement processes related to the forward model is to manipulate the sensorimotor feedback of self‐generated movements. In these experiments, participants typically produce articulatory or manual movements while feedback is altered. For example, studies have investigated how the brain responds to unpredicted feedback by manipulating the acoustic properties of one's own voice (Tourville, Reilly, & Guenther, 2008; Zheng et al., 2013), by introducing illusory visual displacement of the hand or a mechanically controlled avatar (David et al., 2007; Diedrichsen, Hashambhoy, Rane, & Shadmehr, 2005; Schnell et al., 2007), or by applying an unpredicted external physical force (Diedrichsen et al., 2005; Golfinopoulos et al., 2011). Therefore, there can be much variability in how this mechanism is studied in neuroimaging research in terms of form of motor production, feedback manipulation, and sensory modality of feedback. It was our intention to evaluate over the body of literature eliciting activity in response to various manipulations of self‐generated sensory feedback any common areas reported in the brain, with specific interest in finding consensus on the regions of the cerebellum involved. We conducted three activation likelihood estimation (ALE) meta‐analyses of functional neuroimaging studies to identify patterns of neural activation that are reliably affected by these manipulations. A primary analysis was expected to yield a modality independent but anatomically precise global impression of cerebellar contributions to processes related to the forward model. Two secondary ALE analyses were conducted to differentiate this impression in terms of potential modality‐specific components, as well as a contrast analysis between auditory and visual feedback results. This distinction is made as although higher processing cortical areas may be responsive irrespective of the sensory modality, there is cerebellar and cortical distinction between areas responsive to auditory and visuomotor feedback manipulations. In doing so, we may shed light on a consensus of where the cerebellum is involved in processing feedback error, and specifically if the cerebellum is more reliably probed in areas segregated for auditory or visual sensory input. Additionally, due to the high diversity in methods across this body of literature, we aimed to further investigate the dependency of cerebellar activity reported on the factors which most commonly varied across the experiments selected for our meta‐analyses.

## 2 MATERIALS AND METHODS

**Figure 2:**
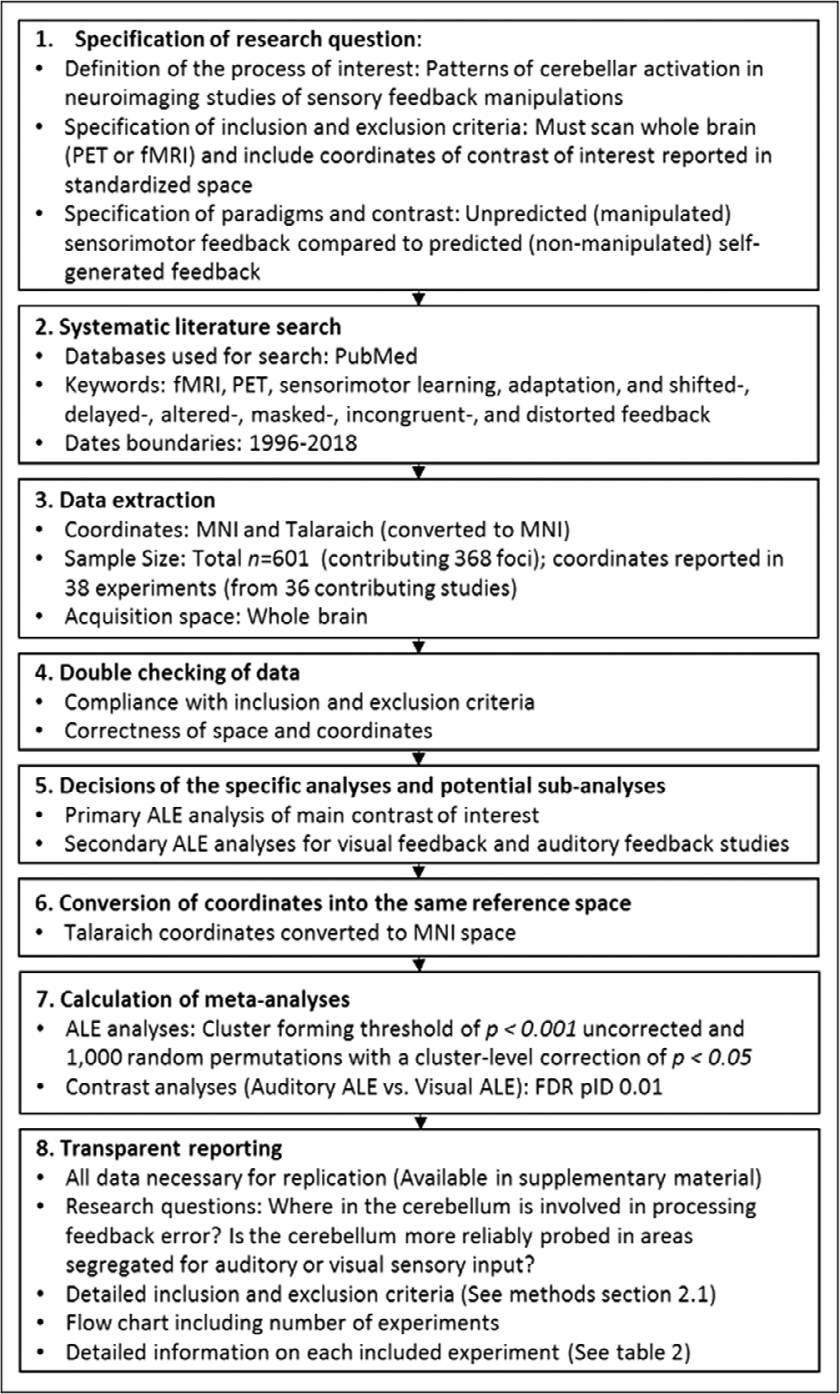
Meta‐analysis flowchart. A flowchart diagram recommended in the best practice guidelines for the conducting of neuroimaging meta‐analyses (Müller et al., 2017). The flowchart lists eight sections. (1) Specification of research question. (2) Systematic literature search. (3) Data extraction. (4) Double checking of data. (5) Decisions of the specific analyses and potential subanalyses. (6) Conversion of coordinates into the same reference space. (7) Calculation of meta‐analyses. (8) Transparent reporting. Each section outlines all relevant information for the study in order to provide the reader with all necessary information for replication

We generally followed recent best practice guidelines for the conducting of neuroimaging meta‐analyses (Müller et al., 2017). These guidelines have been put forward to improve the transparency and replicability of meta‐analyses. We accordingly report information advised such as research question, inclusion and exclusion criteria, detailed information for all experiments, and a step‐by‐step flowchart (see Figure 2).

### 2.1 Study selection

The PubMed (http://www.ncbi.nlm.nih.gov/pubmed) database was searched for human neuroimaging studies using combinations of relevant keywords (e.g., functional magnetic resonance imaging [fMRI], positron emission tomography [PET], sensorimotor learning, adaptation, and shifted‐, delayed‐, altered‐, masked‐, incongruent‐, and distorted feedback). We further cross‐referenced the articles produced from the search term to corroborate that no relevant articles were overlooked. Studies were selected if they reported activation contrasts of unpredicted (manipulated) compared to predicted (non‐manipulated) self‐generated feedback, data acquisition covered the whole brain encompassing the cerebellum, and included tables listing peak activations in standard stereotaxic space. Only data from healthy adult participants were selected. Results reported in Talairach space (Talairarch & Tournoux, 1988) were converted to Montreal Neurological Institute (Holmes et al., 1998) using the GingerALE (2.3.6) icbm2tal conversion algorithm (Lancaster et al., 2007). This search yielded experiments from 36 studies, reporting a total of 368 foci of activation from 601 participants, including 16 studies of manipulated auditory feedback from vocalizations or button‐presses, and 20 studies of manipulated visual feedback of hand movements. Details of the included studies are in Table 1.

**Table 1:** Studies included in meta‐analysis

Study	*N*	Feedback manipulation	Imaging method	Number of foci
Visual feedback				
Anguera, Reuter‐Lorenz, Willingham, and Seidler (2010)	21	Spatial shift	3 T	18
Backasch et al. (2014)	16	Temporal	3 T	6
Balslev, Nielsen, Lund, Law, and Paulson (2006)	15	Temporal	3 T	4
Brand et al. (2017)	14	Spatial shift	3 T	13
David et al. (2007)	14	Mismatch	1.5 T	11
Diedrichsen et al. (2005)	39	Spatial shift	3 T	8
Farrer et al. (2008)	15	Temporal	1.5 T	10
Fink et al. (1999)	10	Mismatch	PET	1
Grafton, Schmitt, Van Horn, and Diedrichsen (2008)	10	Spatial shift	1.5 T	14
Graydon, Friston, Thomas, Brooks, and Menon (2005)	24	Spatial shift	4 T	12
Inoue et al. (2000)	6	Spatial shift	PET	19
Krakauer et al. (2004)	12	Spatial shift	PET	7
Leube et al. (2003)	18	Temporal	1.5 T	12
Limanowski, Kirilina, and Blankenburg (2017)	16	Temporal	3 T	5
Ogawa, Inui, and Sugio (2007)	17	Temporal	1.5 T	3
Seidler, Noll, and Chintalapati (2006)	26	Spatial shift	3 T	12
Schnell et al. (2007)	15	Mismatch	1.5 T	11
Spaniel et al. (2015)	35	Spatial shift	3 T	2
Tunik, Saleh, and Adamovich (2013)	12	Mismatch	3 T	12
Yomogida et al. (2010)	28	Mismatch	1.5 T	6
Auditory feedback				
Behroozmand et al. (2015)	8	Acoustic shift	3 T	16
Christoffels, Firk, and Schiller (2007)	14	Noise mask	3 T	3
Fu et al. (2005)	13	Acoustic shift	1.5 T	6
Golfinopoulos et al. (2011)	13	Physical	3 T	52
Hashimoto and Sakai (2003)	15	Temporal shift	1.5 T	6
Kleber, Zeitouni, Friberg, and Zatorre (2013)	22	Noise mask	3 T	22
McGuire, Silbersweig, and Frith (1996)	6	Acoustic shift	PET	4
Parkinson et al. (2012)	12	Acoustic shift	3 T	6
Pfordresher, Mantell, Brown, Zivadinov, and Cox (2014)	20	Temporal /mismatch	3 T	34
Sakai, Masuda, Shimotomai, and Mori (2009)	10	Temporal	1.5 T	9
Takaso, Eisner, Wise, and Scott (2010)	8	Temporal	PET	4
Tourville et al. (2008)	11	Spatial shift	3 T	18
Toyomura et al. (2007)	12	Spatial shift	1.5 T	6
Zarate and Zatorre (2008)	12	Spatial shift	1.5 T	6
Zheng, Munhall, and Johnsrude (2010)	21	Acoustic shift	3 T	5
Zheng et al. (2013)	20	Acoustic shift	3 T	4

*Note*. Study: included visual and auditory feedback experiments. N: participants from each study contributing to pooled dataset. Feedback manipulation: Visual studies were subject to temporal and spatial shifts, and random mismatch between action and feedback, auditory studies were subject to temporal and acoustic shifts, noise masking, and random mismatch between action and feedback. Imaging method: acquisitions from MRI or PET imaging equipment. Number of foci: amount of contributing foci of significant activity from each study.

Abbreviation: MRI, magnetic resonance imaging.

### 2.2 ALE analyses

The software package GingerALE (2.3.6) was used to perform three analyses on coordinates for peak activations derived from the studies identified by the literature search (http://www.brainmap.org/ale; Eickhoff et al., 2009; Eickhoff, Bzdok, Laird, Kurth, & Fox, 2012; Laird et al., 2005; Turkeltaub et al., 2012). Three single set ALE and one contrast analysis were conducted. ALE computes the likelihood of a voxel for being a source of activation within the set of studies across the whole brain. A primary ALE analysis was conducted on the full dataset of 38 experiments from the 36 studies included, using a cluster forming threshold of *p* < .001 and 1,000 random permutations with a cluster‐level correction of *p* < .05. These parameters were chosen as they were the recommended threshold settings for the cluster‐level multiple test correction listed in the GingerALE software user guidelines. This analysis was performed to identify concordant activations regardless of the feedback modality. A secondary set of ALE analyses was conducted separately on a dataset of 17 experiments from the 16 studies with manipulated auditory feedback, and on 21 experiments from the 20 studies with manipulated visual feedback. Identical cluster forming threshold and correction parameters for the first ALE were applied to the auditory and visual dataset analyses. Contrast analyses were performed comparing auditory and visual ALE results applying a False Discovery Rate assuming independence or positive dependence (FDR pID) of 0.01, with threshold parameters as recommended in the GingerALE manual.

### 2.3 Tests of independence

We investigated the relative success of the most commonly applied experimental designs to engage the cerebellum. To this end, we categorized the studies in our dataset by sensory modality (visual vs. auditory), acquisition methods (blocked vs. event‐related design), response to manipulated feedback (adaption vs. no‐adaptation), and type of feedback manipulation (temporal, spatially or acoustically shifted, masked, mismatched, or physical perturbation). Adaptation studies are categorized as eliciting adjustment in response to sustained manipulation of feedback, where the fine‐tuning of motor commands may be relevant to the success of future action. This differs from automatic compensatory responses to brief changes in feedback that do not inform successive behavior. We tested whether the presence of significant activation within the cerebellum was associated with these methodological factors with a chi‐squared test of independence applying the Yates correction of continuity using SPSS version 24 (IBM Corp., Armonk, NY). Significance thresholds were set at *p* < .05.

## 3 RESULTS

### 3.1 Manipulated feedback: ALE analyses

**Figure 3:**
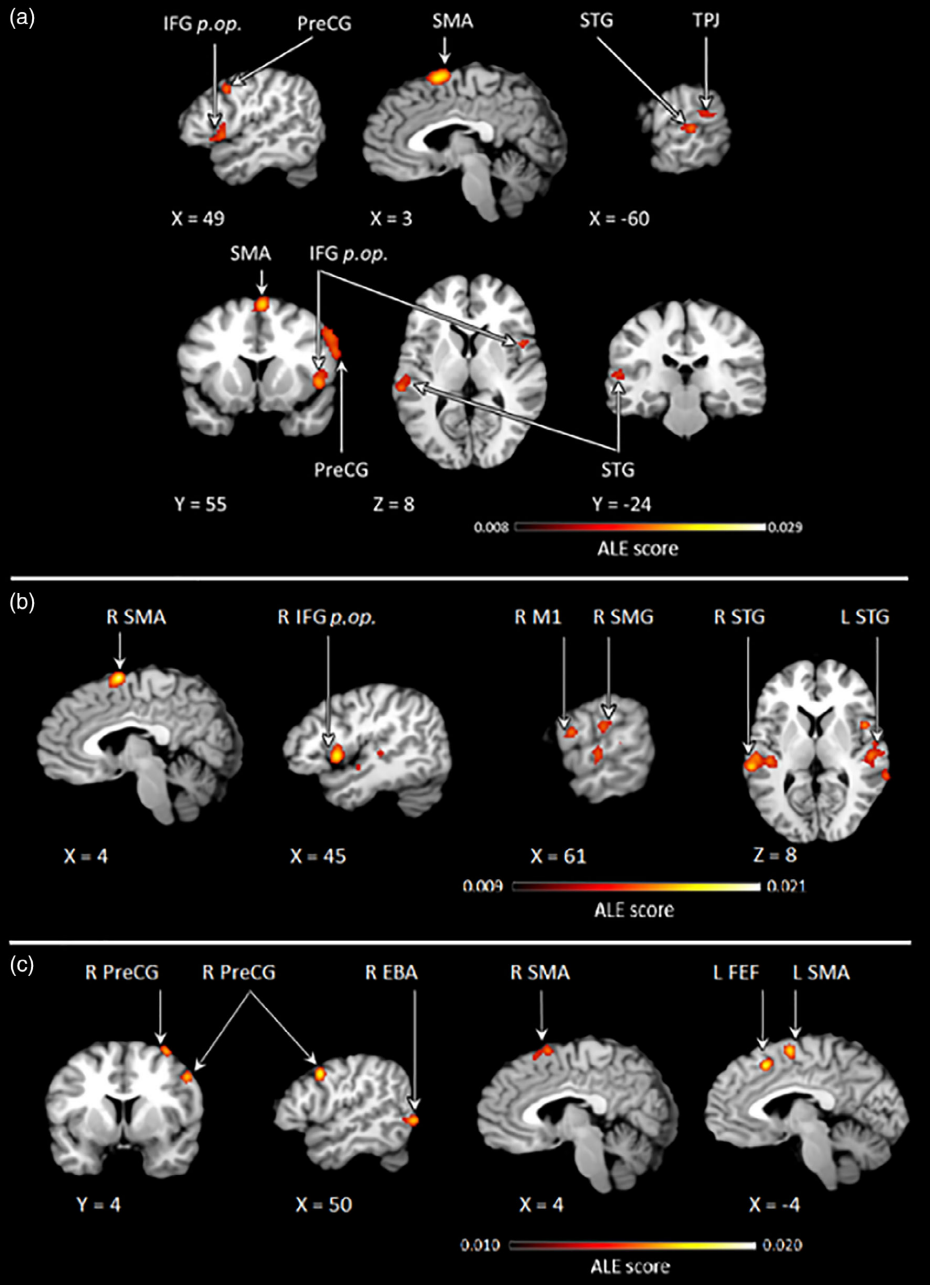
(a) Sensory feedback error ALE. Illustrates results from a meta‐analysis on studies which report data reporting areas of the brain that increase in activity when self‐initiated sensory feedback is experimentally manipulated compared to regular conditions of expected feedback. Images: six slices at MNI space *x* axis 49, 3, −60, *y* axis 55, −24, and *z* axis 8. Units of measurement: ALE scores with a minimum value of 0.008 and maximum of 0.029. A threshold of likelihood calculated from a cluster‐forming threshold of *p* < .001, with a cluster‐level correction of 0.05, 1,000 random permutations. ALE, activation likelihood estimation; L STG, left superior temporal gyrus; L TPJ, left temporoparietal junction; MNI, Montreal Neurological Institute; R IFG p.op., right inferior gyrus pars opercularis; R PreCG, right precentral gyrus; SMA, supplementary motor area. (b) Auditory feedback error ALE. Illustrates results from a meta‐analysis on studies which report data reporting areas of the brain that increase in activity when self‐initiated auditory feedback is experimentally manipulated compared to regular conditions of expected feedback. Images: four slices at MNI space *x* axis 4, 45, 61, and *z* axis 8. Units of measurement: ALE scores with a minimum value of 0.000 and maximum of 0.021. A threshold of likelihood calculated from a cluster‐forming threshold of *p* < .001, with a cluster‐level correction of 0.05, 1,000 random permutations. ALE, activation likelihood estimation; L STG, left superior temporal gyrus; MNI, Montreal Neurological Institute; R IFG p.op., right inferior gyrus pars opercularis; R M1, right primary motor cortex; R SMA, right supplementary motor area; R SMG, right supramarginal gyrus; R STG, right superior temporal gyrus. (c) Visual feedback error ALE. Illustrates results from a meta‐analysis on studies which report data reporting areas of the brain that increase in activity when self‐initiated visual feedback is experimentally manipulated compared to regular conditions of expected feedback. Images: four slices at MNI space *x* axis 50, 4, −4, and *y* axis 4. Units of measurement: ALE scores with a minimum value of 0.010 and maximum of 0.020. A threshold of likelihood calculated from a cluster‐forming threshold of *p* < .001, with a cluster‐level correction of 0.05, 1,000 random permutations. ALE, activation likelihood estimation; L FEF, left frontal eye fields; L SMA, left supplementary motor area; MNI, Montreal Neurological Institute; R EBA, right extrastriate body area; R PreCG, right precentral gyrus; R SMA, right supplementary motor area [Color figure can be viewed at http://wileyonlinelibrary.com]

The primary ALE identified five clusters (Table 2a and Figure 3a). The largest cluster was centered medially in the superior frontal gyrus extending into the right and left supplementary motor area (SMA) (BA 6). There were two right lateralized clusters in the precentral gyrus (preCG) (BA 9) and inferior frontal gyrus (IFG) pars opercularis (BA 44), as well as two left hemisphere clusters in the supramarginal gyrus (SMG) (BA 40) and SMA (BA 6). The secondary ALE on auditory feedback manipulation identified six clusters, centered at the left superior temporal gyrus (STG) (BA 22) and right STG (BA 41), with four additional right lateralized clusters at the SMA (BA 6), IFG pars opercularis (BA 44), SMG (BA 40), and primary motor cortex (M1) (BA 4) (Table 2b and Figure 3b). The secondary ALE on visual feedback manipulation also identified six clusters, centered at the right preCG (BA 6), left hemisphere frontal eye fields (BA 8) and SMA (BA 6), as well as right lateralized clusters at the preCG (BA 6), extrastriate body area (EBA) (BA 37), and SMA (BA 6) (Table 2c and Figure 3c). Remarkably, both the primary and secondary ALE analyses failed to identify clusters of activation in the cerebellum.

**Table 2:** ALE results

Brain regions	BA	MNI coordinates (mm)			Cluster size (mm^3^)
		*x*	*y*	*z*	
A. Sensory feedback error ALE: Manipulated feedback > non‐manipulated feedback					
R/L SMA	6	0.5	3.3	60.3	3,576
R PreCG	9	55.9	9.7	31.7	2,528
R IFG (pars operc.)	44	45.6	13.9	1.2	1824
L STG	42	−57.6	−26	9.2	1,072
L TPJ	22	−61.4	−40.2	19.9	848
B. Auditory feedback error ALE: Manipulated auditory feedback > non‐manipulated auditory feedback					
L STG	22	−56.7	−29.6	14.2	6,400
R STG	41	55.5	−19.3	4.8	4,064
R SMA	6	2.5	3.9	61.3	1,792
R IFG (pars operc.)	44	45.8	9.7	3.8	1,400
R SMG	40	63.8	−21.1	20.3	832
R M1	4	62.3	−.2	18	792
C. ALE visual feedback error ALE: Manipulated visual feedback > non‐manipulated visual feedback					
R PreCG	6	51.5	7.3	38.7	1,000
L FEF	8	−4.5	17.2	46.4	856
L SMA	6	−3.9	−2.4	58	768
R PreCG	6	36.4	1.2	58.4	768
R EBA	37	48.9	−68	2.3	712
R SMA	6	4.6	14	60.1	696

*Note*. ALE analyses: cluster‐forming threshold of *p* < .001 and 1,000 random permutations with a cluster‐level correction of *p* < .05.

Abbreviations: ALE, activation likelihood estimation; L FEF, left frontal eye fields; L SMA, left supplementary motor area; MNI, Montreal Neurological Institute; R EBA, right extrastriate body area; R PreCG, right precentral gyrus; R SMA, right supplementary motor area.

Abbreviations: BA, Brodmann area; EBA, extrastriate body area; FEF, frontal eye fields; IFG, inferior frontal gyrus; M1, primary motor cortex; L, left; PreCG, precentral gyrus; R, right; SMA, supplementary motor area; SMG, supramarginal gyrus; STG, superior temporal gyrus; TPJ, temperoparietal junction.

The subsequent contrast analysis between auditory and visual feedback error ALE results utilized only clusters obtained in at least one of the three individual ALE analyses for comparison. As no cerebellar clusters were produced in either of the ALE analyses, the contrast analysis did not produce any valuable comparisons of modality specific cerebellar clusters. At a cortical level, the auditory compared to visual ALE cluster contrast produced two clusters in the left and right STG specifically for auditory and not visual feedback error. No significant clusters were found for visual and auditory feedback errors nor any common regions between modality in a conjunction of both ALE analyses clusters (see Supplementary Materials Table S1).

### 3.2 Relationship between experimental design and cerebellar activation

To determine whether certain methodological differences were linked with being more likely to activate the cerebellum we conducted chi‐squared tests of independence for four common design features in which experiments differed from one another. With an a priori significance threshold set to *p* < .05, finding cerebellar activation was not contingent on sensory modality (*p* = .243), manipulation type (*p* = .306), or acquisition method (*p* = .071). However, cerebellar activation was contingent on adaptation (*p* = .005). Then, 14 of 18 studies (78%) that elicited adaption responses reported significant activation in the cerebellum. In summary, of the four factors tested, cerebellar activation proved to depend significantly on the need for adaptation.

## 4 DISCUSSION

We conducted three meta‐analyses of neuroimaging studies that altered sensory feedback during ongoing movements with the aim of localizing the cerebellar contributions to processes related to the forward model. In doing so, we attempted to reconcile an apparent lack of consensus of the role of the cerebellum in experiments of unexpected changes to sensory consequences of our own action. Contrary to our expectations and to broad scientific consensus suggesting that the cerebellum is involved in this process, we did not observe a convergence of activation foci in the cerebellum, although the analyses successfully identified the expected network of cortical areas. We therefore systematically assessed methodological factors that could potentially increase the likelihood of specific experimental designs to activate the cerebellum. Our findings confirm that studies that require adaptation of behavior in response to sensory feedback manipulations most reliably evoke cerebellar activation.

### 4.1 Responses to unpredicted feedback

#### 4.1.1 Cerebral cortex

The primary ALE analysis of the manipulated sensory feedback dataset indicated clusters in the SMA, preCG, IFG, STG, and TPJ. Two of the clusters, the SMA and preCG, were centered in the right secondary motor cortex, regions associated with the production of an efference copy for assessing sensory consequences of movement (Christensen et al., 2006; Ellaway, Prochazka, Chan, & Gauthier, 2004; Haggard & Whitford, 2004). The cluster centered at the IFG and extending into the prefrontal frontal regions incorporates areas which lend themselves to a broader self‐awareness network and are thought to reflect agency over action (David, Newen, & Vogeley, 2008; Fink et al., 1999; Jardri et al., 2007; Leube et al., 2003; Nahab et al., 2010). The right IFG plays a role in detecting cues which are relevant to inhibiting motor activity (Corbetta & Shulman, 2002; Hampshire, Chamberlain, Monti, Duncan, & Owen, 2010) and in subsequent reorienting and updating of action plans (Levy & Wagner, 2011). The cluster centered in the TPJ extends into much of the IPL, which is associated with monitoring motor outflow (Desmurget & Sirigu, 2009; Sirigu et al., 1996), as well as with awareness of consistency of intended and actual motor consequences (Farrer et al., 2008), where activity is higher when another agent is active (Decety, Chaminade, Grezes, & Meltzoff, 2002; Farrer & Frith, 2002; Ruby & Decety, 2001). The TPJ has been shown to be active in response to changes in visual and auditory stimuli that are task‐relevant (Downar, Crawley, Mikulis, & Davis, 2001). Almost all clusters identified by the full dataset primary ALE comprised foci from both auditory and visual studies, suggesting a multisensory network. This asserts that some cortical areas process error in both auditory and visual self‐generated feedback. The cluster centered in the left STG however was an exception, with only foci contributed from auditory studies.

Analyses of the auditory subset ALE revealed bilateral STG activation. These auditory cortical processing areas have shown a stronger response to auditory stimuli initiated by others than by oneself (Christoffels et al., 2007; Curio et al., 2000; Heinks‐Maldonado, Mathalon, Gray, & Ford, 2005; Houde, Nagarajan, Sekihara, & Merzenich, 2002; Knolle, Schröger, Baess, & Kotz, 2012; Numminen & Curio, 1999). Increased activation of auditory cortex has also been correlated with the degree of delay in auditory feedback in speech production (Hashimoto & Sakai, 2003) and in response to sound masking compared to expected auditory feedback (Christoffels et al., 2007). Likewise, there was a large medial cluster in the auditory subset in the right SMA. The SMA has been suggested to play a role in auditory sensorimotor associations, for example, in using auditory information to elicit automatic motor responses (Lima, Krishnan, & Scott, 2016) and auditory conditioning in a motor task (Kurata, Tsuji, Naraki, Seino, & Abe, 2000).

Studies that manipulated visual feedback were more likely to activate the premotor cortex of the right preCG, and a cluster in the EBA extending into the IPL. The EBA is involved in the visual processing of perceiving the human body (Downing, Jiang, Shuman, & Kanwisher, 2001; Peelen & Downing, 2007), of goal‐directed action of body parts (Astafiev, Stanley, Shulman, & Corbetta, 2004), and in processing incoherent human biological motion sequences (Downing, Peelen, Wiggett, & Tew, 2006). David et al. (2007) suggest that the EBA may be part of a larger network including posterior parietal cortex, premotor cortex, and the cerebellum, that is involved in correcting sensorimotor discrepancy. The IPL engages in the monitoring and comparison of one's own action and the visual feedback that it generates (Schnell et al., 2007), and in visuomotor incongruencies (Balslev et al., 2006), reported as well more broadly in the parietal cortex (Fink et al., 1999; Shimada, Hiraki, & Oda, 2005).

#### 4.1.2 Cerebellum

Despite a strong consensus suggesting that forward models generally rely on the cerebellum (Bastian, 2006; Ishikawa et al., 2016; Ito, 2005; Kawato & Gomi, 1992; Wolpert et al., 1998), cerebellar activations were reported in only half of the 38 experiments that formed the dataset for the current analyses. The majority of these activations were localized in lobules VI and VIII. Cerebellar lobule VI contains overlapping functional zones that are sensitive to auditory and visual stimulation while a functional zone of lobule VIII is associated with sensorimotor processing (O'Reilly et al., 2009; Sang et al., 2012). The cerebellum receives climbing fiber inputs from the sensory periphery and corticopontocerebellar mossy fiber inputs from cortical motor areas. The cerebellar cortex is arranged into functional modules which act as points of convergence of these inputs (Odeh, Ackerley, Bjaalie, & Apps, 2005). These modules in turn send cerebellothalamocortical projections back up to the corresponding cortical motor areas to inform further movements (Palesi et al., 2017). Through functional connectivity magnetic resonance imaging in humans, there have been efforts to accurately map the functional segmentation of the cerebellum to their corresponding cortical regions (Allen et al., 2005; Habas et al., 2009; Krienen & Buckner, 2009; O'Reilly et al., 2009). For example, Buckner et al. (2011) performed functional connectivity analyses on data from 1,000 participants to provide a comprehensive view of all cerebellar connections with cortex. Their results provide strong evidence for two somatomotor homunculi in the anterior and posterior lobes. The anterior map is centered on lobule VI, while the posterior is centered on lobule VIII. The majority of cerebellar activations in studies included in our analyses were reported in lobules VI (Brand et al., 2017; Diedrichsen et al., 2005; Grafton et al., 2008; Graydon et al., 2005; Inoue et al., 2000; Pfordresher et al., 2014; Tunik et al., 2013; Yomogida et al., 2010; Zheng et al., 2013), and VIII (Anguera et al., 2010; Krakauer et al., 2004; Pfordresher et al., 2014; Tourville et al., 2008). This demonstrates that where cerebellar activations were observed they were not distributed randomly, making these findings unlikely to be false positives.

The majority of activations in the cerebellum reflecting fine‐tuning motor control will be predominately in either of these two somatotopically organized lobules. However, in the ALE analyses, we do not see clustering at each of those regions. The reason for this may be due to the histological organization of the cerebellum. Its structure is completely uniform in its cortex, made up of repeating modules intermixed and overlapping with modules of separate function, having no integral borders (Ito, 1984b). This can lead to a lack of clear separation of focal activity for one specific function. For instance, electrical stimulation at different sites within the cerebellum can induce contraction of the same muscles (Mottolese et al., 2012), while stimulating areas directly adjacent to responsive areas for movement of a body part would cause no movement at all. This creates a difficult picture to deconstruct, where representations in the cerebellum may be very spatially specific, where there may be multiple representations for movement of one body part, and if so where these representations may be sparsely distributed. For these reasons although many studies will report cerebellar activation in response to the same contrast, attempting to find meaningful clusters of functional localization common across studies may be limited.

### 4.2 Considerations for reliable probing of the forward model

Although all studies included in this meta‐analysis contrasted experimental conditions of manipulated feedback with non‐manipulated feedback, there was a considerable degree of diversity in methodology. Experiments varied in terms of feedback modality, the quality and quantity of the feedback manipulation, whether participants were able to adapt behavior, and whether similar experimental trials were blocked together or intermixed.

#### 4.2.1 Choice of feedback manipulation

There was considerable variability in the way that different studies implemented manipulations of sensory feedback from self‐produced action. The most common feedback manipulations employed delays, a mismatch leading to an abrupt loss of control over feedback, noise masking, spectral shifting of auditory feedback or spatial shifting of visual feedback, and the physical application of an external force. The relative amount of cerebellar foci contributing to the study pool from different forms of manipulations differed accordingly. For example, shifted and feedback mismatch studies were twice as likely to elicit cerebellar activity than not (12/18 and 4/6), while temporal manipulations only elicited cerebellar activity in a quarter of the respective experiments (2/8). Among studies that implemented a continuous shift of feedback, six activations were in lobule VI (Brand et al., 2017; Diedrichsen et al., 2005; Grafton et al., 2008; Graydon et al., 2005; Inoue et al., 2000; Zheng et al., 2013), three in lobule VIII (Anguera et al., 2010; Krakauer et al., 2004; Tourville et al., 2008), with two foci just anterior to Lobule VI in the IV/V region (Anguera et al., 2010; Seidler et al., 2006), and two in Crus I/II (Krakauer et al., 2004). The same regions were reported for studies of mismatched feedback, with three foci incorporated in Lobule VI (Pfordresher et al., 2014; Tunik et al., 2013; Yomogida et al., 2010), two in Crus II (Pfordresher et al., 2014; Schnell et al., 2007), and one in Lobule VIII (Pfordresher et al., 2014). However, there was no significant statistical contingency shown between type of manipulation and the report of cerebellar activity in our test of independence analyses.

#### 4.2.2 Motor response or motor disturbance

Feedback manipulations can either elicit an adjustment to feedback or disrupt movement altogether. For instance, there is a tendency to speak louder when auditory feedback is masked (Lane & Tranel, 1971; Lombard, 1911) or to speak more quietly when auditory feedback is amplified (Chang‐Yit, Pick Jr, & Siegel, 1975). In both cases, feedback from the auditory periphery is at an unpredicted level and motor behavior is adapted accordingly. Likewise, by applying an external physical force to speech effectors (Abbs & Gracco, 1984; Gomi, Honda, Ito, & Murano, 2002; Honda, Fujino, & Kaburagi, 2002; Saltzman, Löfqvist, Kay, Kinsella‐Shaw, & Rubin, 1998; Shaiman & Gracco, 2002), or shifting the frequency or pitch of feedback (Donath, Natke, & Kalveram, 2002; Elman, 1981; Houde & Jordan, 1998, 2002; Jones & Munhall, 2000; Larson, Burnett, Kiran, & Hain, 2000; Natke, Donath, & Kalveram, 2003; Purcell & Munhall, 2006; Xu, Larson, Bauer, & Hain, 2004; Zarate, Wood, & Zatorre, 2010; Zarate & Zatorre, 2008), automatic compensation responses are elicited as the speaker attempts to reach their intended auditory targets of their natural sounding speech. These responses of automatic compensation differ from adaptation as they are instantaneous shifts to counteract perturbation, while adaptation can be a conscious process of adjusting motor commands for future behavior in response to continuous change of sensory feedback.

High magnitude manipulations may cause feedback to be perceived as entirely outside a range of control of the actor, and thus no longer triggering an automatic compensation in motor production. This stems from the theory that our sense of agency over sensory feedback from the environment is dependent on the magnitude of discrepancy between the predictable consequences of our own action and the unpredictable external influences of sensory input (David et al., 2008). For example, singers can successfully suppress the automatic compensation response when the pitch of their voice is shifted by a large amount, but not when a smaller shift is applied (Zarate & Zatorre, 2008). The duration of manipulated feedback also influences compensation responses. Pitch shifts with short durations prompted automatic adjustments while pitch shifts with longer durations were more easily ignored (Burnett, Freedland, Larson, & Hain, 1998; Hain et al., 2000; Zarate et al., 2010; Zarate & Zatorre, 2008). Some forms of feedback can disrupt movement altogether. By applying delayed auditory feedback (DAF), speech and musical performance are interrupted (Black, 1951; Fukawa, Yoshioka, Ozawa, & Yoshida, 1988; Havlicek, 1968; Howell & Powell, 1987; Lee, 1950; Mackay, 1968; Siegel, Schork, Pick, & Garber, 1982). The magnitude of delay is also an important consideration, with DAF of approximately 200 ms being the most disruptive (Fairbanks & Guttman, 1958; Hashimoto & Sakai, 2003; Stuart, Kalinowski, Rastatter, & Lynch, 2002). Further increase of delay may lead to similar disregard of feedback as irrelevant to the agency of the actor.

#### 4.2.3 Adaptation to changes in feedback

Unpredicted feedback informs not only adjustments to ongoing movements, but also updates predictions for future movements by means of adaptation. This response to changes in environmental feedback is a form of motor learning and differs qualitatively from motor sequence learning (Doyon, Penhune, & Ungerleider, 2003). This type of motor learning therefor must not be seen as planning new coordinated motor plans, and instead viewed specifically as reoptimization that seeks to minimize future costs to the motor system by forming more accurate predictions of existing motor plans (Izawa, Rane, Donchin, & Shadmehr, 2008). Across the studies in our analyses, cerebellar foci were most common in studies driving adaptation in response to physical perturbation of the mouth (Golfinopoulos et al., 2011) and arm (Diedrichsen et al., 2005), learning new associations between the spatial consequences of movement when visual feedback is shifted (Anguera et al., 2010; Brand et al., 2017; Diedrichsen et al., 2005; Grafton et al., 2008; Graydon et al., 2005; Inoue et al., 2000; Krakauer et al., 2004; Seidler et al., 2006; Zheng et al., 2013), and when vocal pitch was shifted during continuous speech (Tourville et al., 2008; Zheng et al., 2013). All of these manipulations evoke the fine‐tuning of accurately predicting movement outcomes in response to changes in the environment (Ishikawa et al., 2016). Indeed, adaptation was found to be the only factor in our analyses that showed a significant dependency with the elicitation of cerebellar activation.

The cerebellum plays an important role in adapting future predictions in light of error. Cerebellar patients are able to react to changes to feedback (Morton & Bastian, 2006; Smith, Brandt, & Shadmehr, 2000), but are unable adapt by calibrating their predictions for subsequent behavior (Maschke, Gomez, Ebner, & Konczak, 2004; Morton & Bastian, 2006; Smith & Shadmehr, 2005). This suggests that adjustments to feedback, which inform subsequent fine‐tuning, require cerebellar engagement. Monkeys with experimental lesions to areas of the cerebellum which receive mossy fibers from cortex such as the posterior lobe parafloculus and uvula are unable to adapt to changes in feedback (Baizer, Kralj‐Hans, & Glickstein, 1999). Inactivation of deep cerebellar nuclei impairs adaptation to physical and visuomotor perturbation (Kerr, Miall & Stein, 1993). Cerebellar activity may change over time as the system moves from a state of adapting predictions that have failed, to executing predictions that have been adapted (Gilbert & Thach, 1977).

The effect of activation of the cerebellar cortex on adaptation has been reported as well in humans. Cerebellar excitation in transcranial direct current stimulation can lead to faster adaptation to visuomotor feedback transformation (Galea, Vazquez, Pasricha, de Xivry, & Celnik, 2010; Jayaram, Tang, Pallegadda, Vasudevan, Celnik, & Amy Bastian, 2012). Moreover, activity in the cerebellum is greatest immediately after conditions in the environment change (e.g., when feedback is first manipulated) but decreases over time (Friston, Frith, Passingham, Liddle, & Frackowiak, 1992; Nezafat, Shadmehr, & Holcomb, 2001). This has strong implications for the choice of design in experiments seeking to probe the cerebellum's involvement in the forward model.

### 4.3 The implications of fMRI

#### 4.3.1 Neurovascular coupling in the cerebellum

The blood‐oxygen‐level‐dependent (BOLD) response that is measured by fMRI may not be sensitive to some of the neural processes of sensory feedback error in the cerebellum. The BOLD response is correlated with local field potentials (LFP) rather than the spiking rate of neurons (Ekstrom, 2010), which has implications for fMRI studies of the cerebellum. Mossy fiber inputs to the cerebellum synapse thousands of Purkinje cells via parallel fibers and strongly influence LFP, leading to strong increases in the BOLD signal. Climbing fibers communicate via one‐to‐one inputs to Purkinje cells and are thus poorly coupled to the BOLD signal. Experiments that drive cerebellar activity via bottom‐up climbing fiber error signals may be unsuited to measurement by fMRI, whereas experiments that drive cerebellar activity via mossy fiber inputs may lead to detectable BOLD responses (Diedrichsen, Verstynen, Schlerf, & Wiestler, 2010). This is consistent with the view that the mossy fiber input system is more strongly associated with processes of motor learning in adapting to sensorimotor prediction errors (Giovannucci et al., 2017; Ito, 2000; Thach, 1998).

#### 4.3.2 Choice of experimental design

Our findings suggest that some experimental fMRI designs are more appropriately suited to elicit BOLD responses in the cerebellum. Designs that prevent participants from habituating to altered feedback, and continually cause them to adapt their motor responses, may be most effective in eliciting a detectable BOLD response. McGuire et al. (1996) illustrate habituation in block designs as they observed increased activation of the cerebellum during the first half of their study, but not in the latter half. This is consistent with the broader finding that the cerebellum may be more strongly engaged in adjusting to altered feedback than applying adjustments that have already been computed (Andersson & Armstrong, 1987; Flament, Ellermann, Kim, Uǧurbil, & Ebner, 1996; Horn, Pong, & Gibson, 2004; Imamizu et al., 2000; Moberget, Gullesen, Andersson, Ivry, & Endestad, 2014). However, the arrangement instead of fast cycling between trials of different conditions in event‐related designs may hinder adaptation responses if feedback is not consistent from trial to trial. Long events of consistent perturbation (e.g., Christoffels et al., 2007; Grafton et al., 2008; Limanowski et al., 2017) or similarly with short blocks (e.g., Inoue et al., 2000; McGuire et al., 1996; Seidler et al., 2006) may be best suited for probing processes related to the forward model associated BOLD response in the cerebellum.

## 5 CONCLUSIONS

We performed three ALE meta‐analyses and one contrast analysis of functional neuroimaging studies that manipulated predicted self‐initiated auditory, visual, and sensory feedback with the primary aim to identify cerebellar areas responsive to prediction error. No cerebellar clusters were produced as a result of these analyses. Contrary to common presumptions, we found that not all studies that used such approach show significant activation of the cerebellum, as well as variability in where in the cerebellum activations were reported. Our study suggests that this discrepancy stems from differential sensitivity and specific limitations of the experimental paradigms employed across MR neuroimaging altered sensory feedback experiments. These method‐specific characteristics can restrict compatibility with other frameworks, which overwhelmingly support the involvement of the cerebellum in responding to errors in predicted feedback as part of the forward model. We therefore assessed methodological variations that may determine the success of brain imaging experiments in evoking cerebellar activation. The results indicate that experimental designs which most reliably evoked cerebellar activation employed continuous feedback manipulations relevant for adapting motor plans for future action. Due to constraints of neurovascular coupling in cerebellar activity, it is possible that only mossy fiber inputs in response to adaptation elicit demonstrable BOLD signals, while error signals conveyed via climbing fiber spike firing increase may not suitable for fMRI testing. The results further suggest that short‐blocked designs may offer the most effective approach, engaging a period of adaptation to changes in feedback without reaching a state of habituation, leading to reliable activation of the cerebellum.

## CONFLICT OF INTEREST

The authors state no conflict of interest.

## Supporting information


**Supplementary Table SI** Contrast Results

## Data Availability

The data that support the findings of this study are available from the corresponding author upon reasonable request.
