# Supplementary material for: The role of the cerebellum in adaptation: ALE meta‐analyses on sensory feedback error
Source: Hum Brain Mapp. 2019 Jun 2;40(13):3966–81. doi: 10.1002/hbm.24681 (PMC6771970; doi:10.1002/hbm.24681)
Supplement: Supplementary file 1 — Supplementary Table SI Contrast Results [file HBM-40-3966-s001.docx]

| **Supplementary Table I. Contrast Results** | | | | | | | | | |
| --- | --- | --- | --- | --- | --- | --- | --- | --- | --- |
|  | |  |  |  | MNI Coordinates (mm) | | |  | Cluster Size |
| Brain Regions | BA | | |  | x | y | Z |  | (mm^3) |

| **A. Auditory Feedback Error > Visual Feedback Error:** | | | | | | | | |
| --- | --- | --- | --- | --- | --- | --- | --- | --- |
| L STG (A1) | | 41 |  | -59.9 | -28.2 | 10.7 |  | 4456 |
| R STG (A1) | | 41 |  | 53.9 | -16.3 | 4 |  | 2528 |
| **B. Visual Feedback Error > Auditory Feedback Error** | | | | | | | | |
| - |  | - |  | - | - | - |  | - |
| **C. Auditory Feedback Error ᴖ Visual Feedback Error:** | | | | | | | | |
| - | | - |  | - | - | - |  | - |

BA, Brodmann area; L, left; R, right; STG, superior temporal gyrus; A1, primary auditory cortex

Contrast Analyses: Using ALE clusters reported in Auditory Feedback Error ALE (Table II B) and Visual Feedback Error ALE (Table II C) with (cluster-level inference threshold correction (P<0.05) with a cluster-forming threshold of uncorrected p<0.001 using 1,000 permutations). Contrast thresholding with False Discovery Rate (pID) 0.01.
